# Effect of minimally invasive caries management with silver diammine fluoride on caries progression and subsequent need for dental general anaesthesia in children: a retrospective cohort study

**DOI:** 10.1007/s00784-026-07277-w

**Published:** 2026-11-14

**Authors:** Ruba Abdulrahim, Christian H. Splieth, Marcus Vollmer, Manasi R. Khole, Ruth M. Santamaría

**Affiliations:** 1 Department of Pediatric Dentistry, University Medicine Greifswald, D-17475 Greifswald, Germany; 2 Institute of Bioinformatics, University Medicine Greifswald, Greifswald, D-17475 Germany

**Keywords:** Silver diammine fluoride, Paediatric dentistry, Dental caries, Dental general anaesthesia, Minimally invasive treatment, Primary teeth

## Abstract

**Objectives:**

Silver diammine fluoride (SDF) has been reintroduced into paediatric dentistry for managing caries in high-risk patients. This study aimed to describe patient- and tooth-level outcomes following SDF-initiated caries management in children with early childhood caries at a German university dental centre. The primary outcome was subsequent dental general anaesthesia (DGA), and the secondary outcome was tooth-level clinical failure.

**Materials and methods:**

Clinical data were retrospectively extracted from the records of children aged 1–8 years who received 38% SDF between 2019 and 2020. Included patients had a high caries burden and/or limited cooperation with invasive chairside treatment. Tooth-level failure was classified as minor (reversible pulpitis or documented caries progression) or major (irreversible pulpitis or abscess). Patient-level outcomes were analysed descriptively. Tooth-level outcomes were evaluated using Kaplan–Meier survival analysis and Cox proportional hazards regression, accounting for the clustering of multiple teeth within children.

**Results:**

A total of 52 patients (mean age 4.0 ± 1.5 years; mean dmft = 7.8 ± 3.7) with 304 SDF-treated primary teeth (ICDAS 3–5 lesions) were included. After a mean follow-up of 23.4 ± 12.5 months, 46 of 52 children (88.5%) were managed without DGA, while 6 (11.5%) underwent DGA. At the tooth level, 273 teeth (89.8%) remained clinically successful, whereas 31 teeth (10.2%) developed a major failure. No minor failures were documented. Of the teeth without major failure, 165 received no further restorative treatment, while 108 underwent subsequent minimally invasive restorative treatment as part of the staged treatment pathway.

**Conclusion:**

In this retrospective cohort, an SDF-initiated, minimally invasive treatment pathway was associated with favourable patient- and tooth-level outcomes. Initial caries stabilisation may allow time for cooperation to improve and subsequent restorative treatment to be performed without DGA. Comparative prospective studies are needed to determine the specific contribution of SDF.

**Clinical relevance:**

An SDF-initiated treatment pathway may support caries management in young children with a high caries burden or limited cooperation and may help postpone or potentially avoid DGA.

## Introduction

Early childhood caries (ECC) remains a major global health burden, affecting approximately 48% of young children worldwide [1]. Management is often challenging due to limited cooperation and extensive treatment needs [2]. In such cases, DGA is frequently required to enable comprehensive dental treatment in children unable to tolerate conventional chairside care. However, given the associated risks and substantial healthcare burden, its use should be restricted to cases with clear clinical indications. This underscores the need for effective, minimally invasive alternatives that may reduce reliance on DGA.

Dental general anaesthesia (DGA) has been increasingly utilised in paediatric dentistry and represents an essential treatment modality for selected patient groups [3]. Young patients who cannot tolerate conventional chairside treatment or lower-risk conscious sedation techniques such as nitrous oxide inhalation are often referred for treatment under DGA. However, concerns have been raised regarding potential overuse of DGA and inappropriate referrals [4], especially since DGA carries inherent systemic risks associated with general anaesthesia, including respiratory depression, cardiovascular complications, and allergic reactions that could be fatal [5–7]. In addition, the possible neurodevelopmental effects of early or prolonged exposure to DGA have been debated, prompting a safety warning from the U.S. Food and Drug Administration (FDA) [8]. Furthermore, patients undergoing treatment under DGA typically experience longer postoperative recovery times, as well as common adverse effects such as post-operative oral pain, nausea, and dizziness [9]. Alongside the increased costs and resource demands, DGA places a substantial financial and logistical burden on healthcare systems due to the need for specialised equipment, facilities, and trained personnel [10]. Consequently, extended waiting times for DGA appointments have become a frequent source of frustration for parents and caregivers [11]. Collectively, these challenges represent significant barriers to timely and accessible dental care for children.

Silver diammine fluoride (SDF) has been thoroughly investigated and is widely recognised as an effective strategy for caries prevention and management in both primary and permanent dentitions. High-level evidence, including systematic reviews with meta-analyses [12–14] and an umbrella review [15], consistently supports its efficacy and clinical applicability.

Given its well-established efficacy in arresting carious lesions, SDF may represent a potential approach to reducing the need for DGA in children with ECC. Owing to its minimally invasive nature and sustained cariostatic effect, SDF can stabilise disease progression and serve as an interim management strategy. This may facilitate behavioural adaptation and improve cooperation over time, enabling treatment in a conventional chairside setting. However, whether SDF use is associated with a reduced need for DGA remains largely unexplored in clinical practice. Therefore, the present study aimed to describe the subsequent need for dental general anaesthesia (DGA) and tooth-level clinical outcomes in children with early childhood caries treated with 38% SDF at a German university dental centre and to explore factors associated with subsequent DGA.

## Materials and methods​

### Ethical approval and study design

Ethical approval for this study was obtained from the Research Ethics Committee of the University of Greifswald (protocol number BB-142/20). This retrospective cohort study was based on routinely collected clinical data derived from a comprehensive chart review of dental records from patients treated at a German university hospital.

### Inclusion and exclusion criteria

Children aged 1 to 8 years with early childhood caries (ECC) and/or multiple carious lesions in primary dentition with high treatment needs were included. Eligible children were those considered at risk for dental general anaesthesia (DGA) due to extensive caries experience and limited cooperation during chairside treatment.

Patients were included if they met the following criteria:

- Receipt of referral form for DGA by an external referring dentist or an in-house paediatric dentist.
- Assessment by a departmental paediatric dentist indicating ECC and/or extensive caries experience (dmft exceeding the child’s chronological age) and low cooperation, defined as a Frankl Behaviour Rating Scale score of 1 (definitely negative) or 2 (negative).
- Receipt of 38% silver diammine fluoride (SDF) as part of their clinical management.
- Attendance at follow-up visits two years after the initial SDF application.

Patients with clinical signs or symptoms suggestive of irreversible pulp involvement at baseline were excluded, as well as those with a history of DGA or prior SDF application before the study period, to minimise potential confounding of treatment outcomes. Additionally, children with medical or developmental conditions known to substantially affect oral health status, caries progression, or cooperation during dental treatment (e.g., immunodeficiencies or relevant congenital syndromes) were excluded, in order to reduce clinical heterogeneity and potential confounding.

Cases referred for DGA for non-caries-related reasons (e.g., dental trauma) were also excluded. Patients with incomplete or insufficiently documented records were excluded to ensure data quality and reliability.

### Outcome measures

1. Primary outcome: Subsequent DGA
  Patients were followed up for up to two years after the index visit, and the occurrence of any dental treatment performed under general anaesthesia during this period was recorded.

2. Secondary outcome: Tooth-level clinical outcome
  Treated teeth were recorded and assessed for clinical success or failure based on predefined criteria, adapted from Innes et al. and Santamaría et al. [16, 17], to evaluate the effectiveness of SDF in arresting carious lesions.

A minor failure was defined as the presence of signs or symptoms consistent with reversible pulpitis or evidence of caries progression. A major failure was defined as the presence of signs or symptoms indicative of irreversible pulpitis or dental abscess formation (Table 1).

**Table 1:** Clinical criteria for assessing treatment outcomes of SDF-treated teeth

Outcome category	Clinical criteria
Success	• Arrested caries lesion (lesion surface hard on gentle probing) with characteristic SDF-related black staining. • Tooth restored or left unrestored without clinical signs of caries progression. • Absence of clinical symptoms indicating irreversible pulpal pathology (e.g., pain, swelling, or fistula). • Tooth exfoliated naturally without evidence of failure.
Minor Failure	• Evidence of caries progression (soft or leathery lesion on probing, partial or absent SDF-related staining). • Signs or symptoms consistent with reversible pulpitis, managed conservatively without the need for pulp therapy or extraction.
Major Failure	• Clinical signs or symptoms indicative of pulpitis (e.g., spontaneous or persistent pain) requiring pulp therapy or extraction. • Presence of dental abscess or other signs of pulpal necrosis requiring pulp therapy or extraction.

*SDF*, silver diammine fluoride

### Treatment overview

The study sample comprised patients referred from external dental practices as well as new patients seeking care at the University Department of Pediatric Dentistry. This specialised department functions as a regional referral centre within the framework of the German healthcare system, which fully covers all essential dental treatments, including preventive, restorative, and surgical procedures performed either chairside or under DGA. The mean caries experience among children treated at this centre is substantially higher than national averages, with reported dmft values up to five times higher than reference data for Germany [18]. As part of the standard protocol for new or referred patients, initial appointments include a comprehensive clinical examination and individualised preventive care, which includes oral hygiene instructions and fluoride varnish application, followed, when indicated and feasible, by radiographic assessment; this protocol follows the standards of the German healthcare system. Basic behaviour management techniques (e.g., Tell-Show-Do, distraction, and positive reinforcement) are applied to support patient cooperation, which is assessed at each visit using the Frankl Behaviour Rating Scale. Following diagnosis, legal guardians are informed of available treatment options, and a treatment plan is developed in agreement with them, considering modalities such as chairside treatment, nitrous oxide sedation, or DGA, depending on the child’s clinical condition and level of cooperation.

For children presenting with ECC and/or multiple asymptomatic carious lesions in the primary dentition, 38% SDF may be considered for caries management as an off-label treatment because, in Germany, the available product is approved for dentinal hypersensitivity but not specifically for dental caries. With informed parental consent, SDF is applied according to the manufacturer’s instructions and light-cured [19]. Afterwards, patients are managed according to their individual treatment needs and cooperation levels, with recall visits scheduled two to four times per year. In cases of irreversible pulpitis, dental abscess, or acute pain, where SDF application is contraindicated, other treatment modalities are selected according to clinical indications. All treatment procedures are performed by paediatric dental specialists or postgraduate students in paediatric dentistry under direct specialist supervision. Additionally, all clinicians received training and adhered to the manufacturer’s instructions for SDF use.

### Data extraction and statistical analysis

The patient cohort was identified through the department’s electronic record system (DAMPSOFT^®^). Clinical records of children treated between 2019 and 2020 were systematically reviewed, with follow-up extending until the end of 2022. As retrospective studies are regularly conducted at this centre, patient information is documented in a standardised electronic format using predefined fields to ensure data consistency and comparability. All clinicians received structured training in data entry protocols to enhance the accuracy and reliability of clinical documentation. Data were collected and decoded by the main investigator (RA) and reviewed by at least one co-investigator using a Microsoft Excel (2020) spreadsheet prepared for the purpose of this study.

Extracted data included demographic characteristics (age, sex, health status), baseline clinical findings (dmft and clinical diagnosis), child cooperation, and the number of teeth treated with SDF. For analysis, age was categorised into < 6 years and ≥ 6 years. For the descriptive patient-level presentation, cooperation was dichotomised as negative (Frankl scores 1–2) or positive (Frankl scores 3–4) to improve interpretability.

Statistical analyses were performed using R (version 4.5.1; R Foundation for Statistical Computing, Vienna, Austria). Descriptive statistics were used to summarise patient characteristics and baseline clinical variables. Continuous variables are presented as means and standard deviations or medians and interquartile ranges, as appropriate, and categorical variables as frequencies and percentages.

The primary outcome was subsequent DGA during the follow-up period. This outcome was analysed descriptively at the patient level by reporting the number and proportion of children who underwent DGA and the time from SDF application to DGA. Given the small number of children who subsequently underwent DGA (*n* = 6), comparisons according to DGA status were limited to descriptive analyses. No formal hypothesis tests or regression analyses were performed because of the limited statistical power and the risk of unstable estimates.

The secondary outcome was tooth-level clinical failure following SDF treatment. Time-to-event analyses were performed using Kaplan–Meier survival estimates, with observations right-censored at the end of follow-up or at the time of tooth exfoliation. Differences between groups were assessed using the log-rank test. In the tooth-level Cox regression, the four original Frankl categories were retained to preserve the information provided by the scale and to explore differences between individual levels of cooperation. Cox proportional hazards regression models were applied to identify factors associated with tooth-level failure. Given the hierarchical structure of the data (multiple teeth within children), clustering at the patient level was accounted for using robust standard errors. Hazard ratios (HRs) are reported with 95% confidence intervals (CIs). The proportional hazards assumption was assessed prior to model interpretation.

Patients without any follow-up data were excluded, while those with partial follow-up were retained and analysed according to available data. Missing data were handled using complete-case analysis. All statistical tests were two-tailed, and a p-value < 0.05 was considered statistically significant.

## Results

### Baseline characteristics

**Fig. 1:**
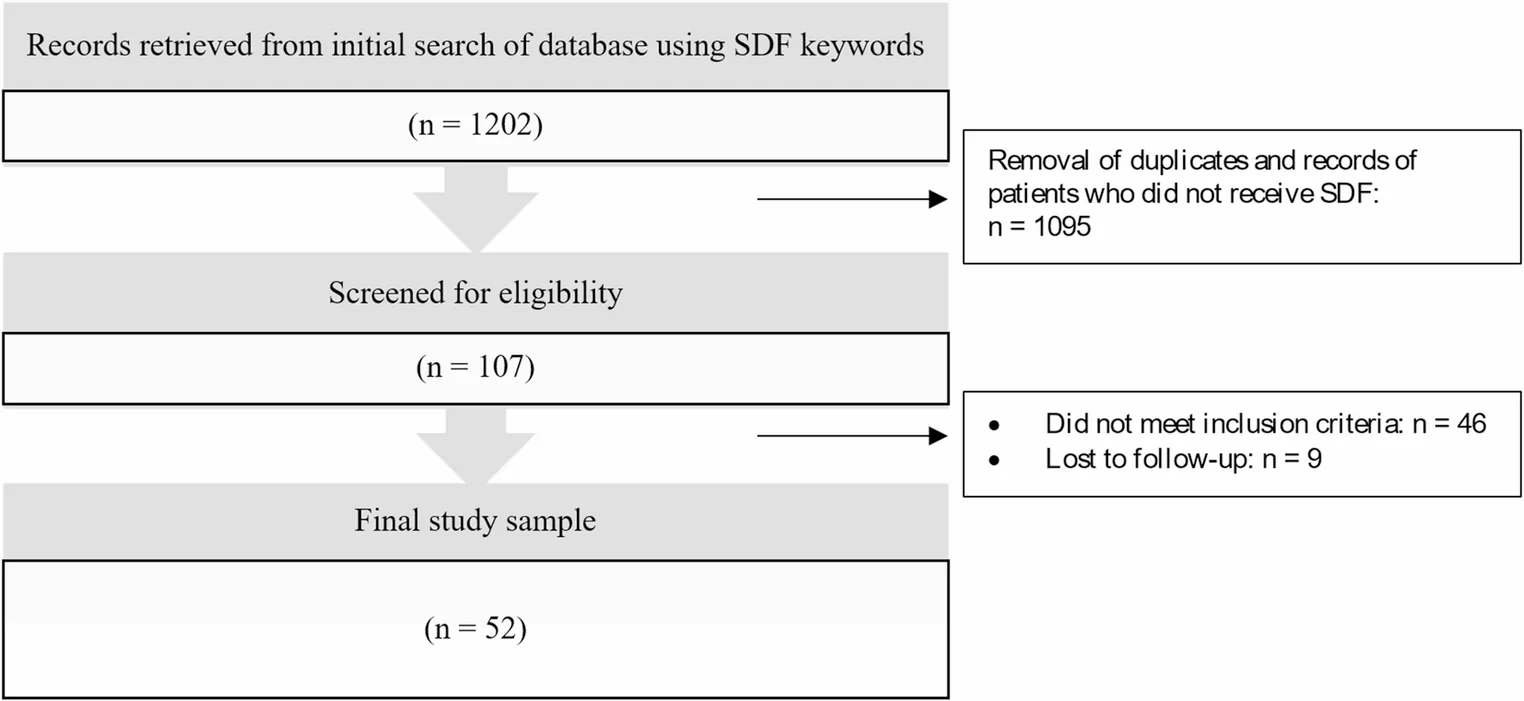
Flow chart of the retrieval process of the study sample

A total of 52 patients (mean age of 4.0 ± 1.5 years) with a mean dmft index of 7.8 ± 3.7 were included in the study, contributing to a total of 304 carious SDF-treated teeth. Of these, 41.8% (*n* = 127) were anterior and 58.2% (*n* = 177) posterior primary teeth, presenting with ICDAS 3–5 lesions without clinical or radiographic signs of irreversible pulpal involvement. Treatment was provided by 11 clinicians in the department: four paediatric dental specialists and seven postgraduate paediatric dentistry trainees. Of the 52 patients, 31 (59.6%) were treated by postgraduate trainees and 21 (40.4%) by paediatric dental specialists. Figure 1 shows the retrieval process of the study sample.

### Follow-up: patient-level outcomes

Follow-up treatment-related descriptive characteristics at the patient level are presented in Table 2. After a mean follow-up of 23.4 ± 12.5 months, 46 of 52 children (88.5%) were managed without DGA, while six (11.5%) underwent DGA. The mean time to DGA was 11 ± 11 months, while the median time was 5 months (IQR: 4–24), reflecting substantial variability in time to event. In contrast, children who did not undergo DGA remained stable for a mean follow-up of 27 ± 9 months (IQR = 19–34).

**Table 2:** Descriptive patient-level characteristics according to subsequent DGA status

Characteristic	Failure (Subsequent DGA; n = 6)	Success (non-DGA; n = 46)
Time to event / follow-up^a^ (months), mean (SD)	11 (11)	27 (9)
Age (years), mean (SD)	4.12 (0.63)	4.00 (1.56)
dmft, mean (SD)	7.2 (2.3)	7.9 (3.8)
Cooperation level, n (%)		
* Negative (Frankl 1–2)*	6 (100)	28 (60.9)
* Positive* (*Frankl 3–4)*	0 (0.0)	18 (39.1)
Teeth treated with SDF, mean (SD)	7.83 (1.72)	5.59 (2.98)
Number of SDF applications, n (%)		
* 1*	4 (66.6)	32 (69.6)
* 2*	1 (16.7)	10 (21.7)
* 3*	0 (0.0)	3 (6.5)
* 4*	1 (16.7)	1 (2.2)
Operator type, n (%)		
* Postgraduate student*	3 (50.0)	28 (60.9)
* Paediatric dental specialist*	3 (50.0)	18 (39.1)

^a^For the failure group, values represent time to dental general anaesthesia; for the success group, values represent duration of follow-up

*SD*, standard deviation; *Frankl*, frankl behaviour rating scale; *SDF*, silver diammine fluoride; *dmft*, decayed, missing, and filled teeth; *DGA*, dental general anaesthesia

Children in the failure (DGA) and success (non-DGA) groups were descriptively similar in age (4.1 ± 0.6 vs. 4.0 ± 1.6 years) and caries experience (dmft: 7.2 ± 2.3 vs. 7.9 ± 3.8). However, cooperation differed between groups: all children in the failure group (100%) exhibited negative behaviour (Frankl scores 1–2) at the first follow-up, compared with 60.9% in the success group, in which 39.1% demonstrated positive cooperation.

The number of teeth treated with SDF was higher in the failure group (7.8 ± 1.7) than in the success group (5.6 ± 3.0). The distribution of SDF applications was similar between groups, with the majority of children receiving a single application (failure: 66.7%; success: 69.6%). Operator type did not differ substantially between groups.

#### Follow-up: tooth-level clinical outcomes

Of the 304 SDF-treated teeth, 31 (10.2%) were classified as major failures, while 273 (89.8%) were successfully managed. No minor failures were observed.

Molars accounted for the majority of major failures (80.6%), followed by incisors (19.4%), whereas no failures were observed in canines.

Regarding subsequent management, teeth classified as major failures were predominantly extracted (87.1%), while the remaining 12.9% received pulp therapy, including pulpotomy and placement of a preformed metal crown (PMC).

Among successfully treated teeth, 108 (39.6%) underwent further restorative management within a minimally invasive treatment approach, including the Hall Technique, direct restorations, or glass ionomer-based techniques (SMART-Silver Modified Atraumatic Restorative Treatment). The remaining 165 teeth (60.4%) remained asymptomatic and did not receive any additional restorative intervention during follow-up (Table 3).

**Table 3:** Tooth-level clinical outcomes and subsequent treatments following SDF application

Outcome	*n* (%)	Subsequent Treatment	*n* (%)
Success	273 (89.8)	No further treatment/regular follow-up	165 (54.3)
		Further restorative treatment	108 (35.5)
		*Hall Technique*	61 (20.1)
		*Direct fillings (Composite/Compomer)*	31 (10.2)
		*Glass ionomer restorations*	14 (4.6)
		*Anterior strip crowns*	2 (0.6)
Minor failure	0 (0.0)	Caries progression/secondary caries	0 (0)
Major failure	31 (10.2)	Irreversible pulpitis/abscess	31 (10.2)
		*Extraction*	27 (8.9)
		*Pulpotomy and PMC*	4 (1.3)
Total	304 (100)	-	-

*PMC*, preformed metal crown

### Survival and multivariate analysis

**Fig. 2:**
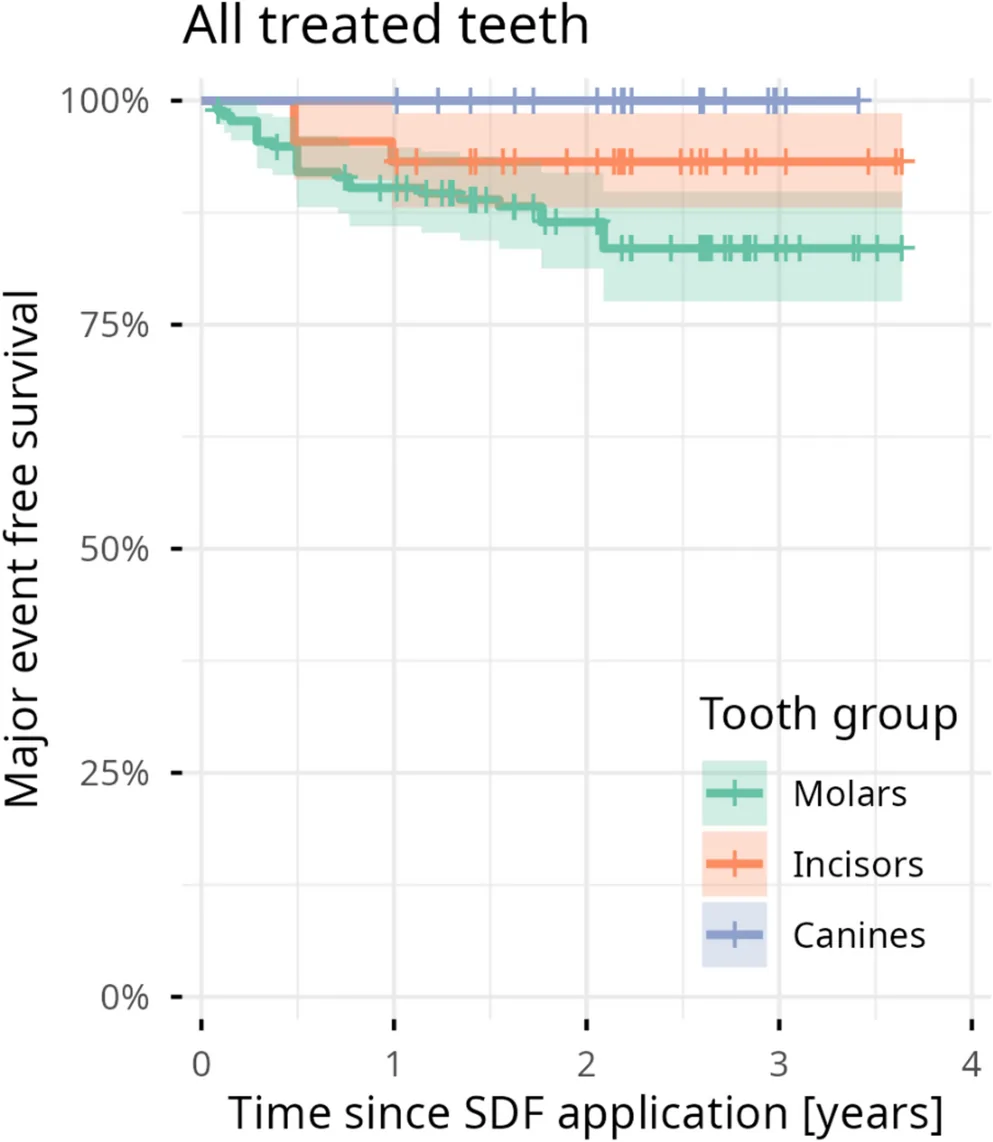
Kaplan-Meier survival curves for tooth level outcomes (absence of failure), stratified by tooth type

Tooth-level survival analysis demonstrated a high probability of survival following SDF treatment over the observation period. Due to the low number of failure events, median survival time was not reached. A statistically significant difference in survival was observed between anterior and posterior teeth (log-rank test, *p* = 0.009), with posterior teeth showing a higher risk of failure. Figure 2 shows the comparison of Kaplan-Meier survival curves at the tooth level for overall success (absence of failure), stratified by tooth type.

Cox proportional hazards regression analysis (Table 4) identified several factors associated with treatment failure following SDF application. Compared with molars, both incisors (HR = 0.10; 95% CI 0.04–0.24; *p* < 0.001) and canines (HR = 0.38; 95% CI 0.20–0.75; *p* = 0.005) showed a significantly lower hazard of failure, indicating higher survival probabilities.

**Table 4:** Cox regression analysis for predictors of treatment failure of SDF-treated teeth (*n* = 304)

Characteristic	*N*	HR (95% CI)	*p*-value
Tooth			
*Molars*	177	—	
*Incisors*	88	0.10 (0.04–0.24)	< 0.001
*Canines*	39	0.38 (0.20–0.75)	0.005
Age categories			
*<6 years*	273	—	
*≥6 years*	31	0.27 (0.08–0.99)	0.048
Operator			
*Postgraduate master student*	188	—	
*Paediatric dental specialist*	116	1.76 (0.93–3.33)	0.081
*Cooperationa*			
*Definitely negative (Frankl 1)*	100	—	
*Negative (Frankl 2)*	112	0.78 (0.40–1.52)	0.47
*Positive (Frankl 3)*	49	0.42 (0.14–1.24)	0.12
*Definitely positive (Frankl 4)*	43	0.69 (0.25–1.92)	0.48

*HR*, Hazard Ratio; *CI*, Confidence Interval; ^a^ Cooperation was entered into the Cox regression as a four-level categorical variable, with Frankl score 1 (definitely negative) as the reference category

Older children showed a significantly lower failure risk compared with younger children (HR = 0.27; 95% CI 0.08–0.99; *p* = 0.048).

Operator type was not significantly associated with treatment outcome in the overall analysis (HR = 1.76; 95% CI 0.93–3.33; *p* = 0.081). In an exploratory subgroup analysis restricted to molars in older children, a higher hazard of failure was observed for teeth treated by paediatric dental specialists compared with postgraduate students (HR = 3.48; 95% CI 1.88–6.43; *p* < 0.001). This finding may reflect differences in case complexity.

Behavioural cooperation and patient origin were not significantly associated with tooth survival (all *p* > 0.05). Overall, tooth type and age emerged as the primary factors associated with treatment outcomes, with molars and younger age linked to a higher risk of failure.

## Discussion

Evidence from controlled clinical trials has supported the efficacy of silver diammine fluoride (SDF) in caries management; however, its potential role within treatment pathways aimed at reducing or postponing dental general anaesthesia (DGA) in children remains insufficiently explored. In the present cohort, a high proportion of children (88.5%) were managed without DGA over a follow-up period of approximately two years. This finding is clinically relevant given the well-documented risks, costs, and logistical challenges associated with DGA [3–7].

The global prevalence of untreated caries in primary teeth remains alarmingly high, affecting over 48% of children worldwide [10]. Significant gaps in care persist, even in countries with well-organised healthcare systems such as Germany, where a broad spectrum of dental services, including preventive, restorative, and surgical treatment modalities, are fully covered for children. According to the most recent German National Oral Health Survey (DAJ, 2017), the care index (proportion of treated to total carious teeth) is only 26% among 3-year-olds and 57.5% among 6- to 7-year-olds [20]. These findings indicate that, despite comprehensive healthcare coverage and established preventive frameworks, a substantial proportion of preschool children with caries remain untreated.

Managing young children with extensive caries remains a major clinical challenge, often requiring behavioural or pharmacological support, including DGA [11]. In Germany, nearly half of all general anaesthesia procedures in children under five are performed for dental indications [21]. Consequently, current guidelines recommend a stepwise approach, prioritising non-invasive and minimally invasive strategies such as behavioural techniques, conscious sedation, and caries control measures before considering DGA [21]. In this context, the present study evaluated clinical outcomes following the use of 38% SDF in a high-risk cohort of pre-cooperative preschool children (mean dmft ≈ 8). Despite the high disease burden, the majority of treated teeth (273 of 304; 89.8%) remained clinically successful over a two-year follow-up, while 31 teeth developed major failures, including irreversible pulpitis or abscess. Failures tended to occur early (median time to failure = 5 months), suggesting that major failures become evident within the initial phase following SDF application.

At the patient level, baseline age and caries experience were descriptively similar between children who subsequently required DGA and those who were managed without DGA. Because only six children underwent DGA, no formal patient-level comparisons were performed, and these observations should not be interpreted as evidence of equivalence between the groups. The higher mean number of SDF-treated teeth in the DGA group (7.8 vs. 5.6) may indicate a greater overall treatment burden; however, this descriptive finding requires confirmation in larger studies. Furthermore, operator type was not associated with treatment success no evidence of an association between operator type and tooth-level failure was observed, suggesting that SDF can be implemented by clinicians with different levels of experience when appropriate training and specialist supervision are provided [22]. However, a possible operator effect cannot be excluded because of the limited sample size and number of failure events.

Previous studies have suggested that repeated SDF applications may influence treatment outcomes [23, 24]. In the present study, distribution of application frequency was descriptively similar between the DGA and non-DGA groups, with most children receiving a single application (DGA: 66.6%; no DGA: 69.6%). However, because only six children experienced failure and no statistical between-group analysis was performed, no firm conclusion can be drawn regarding the association between application frequency and treatment outcomes. Notably, no documented minor failures were observed. Lesions managed without further restorative intervention were considered clinically arrested, while additional restorations were performed as part of a staged treatment approach after patient cooperation had improved rather than because of documented caries progression. This finding may reflect not only the SDF application but also the structured preventive care available within the German healthcare system, which provides regular, risk-based preventive interventions, including fluoride varnish applications, professional cleaning, and dietary counselling, up to four times per year. These findings are consistent with previous reports, including an Australian clinical trial in which 88% of high-risk children were managed without DGA following a single SDF application over a 6-month follow-up period [25]. Given that dental caries is a controllable disease, the long-term success of an SDF-based treatment pathway may depend on sustained preventive care. Accordingly, reinforcing parental and child awareness of oral health behaviours remains essential to maintaining treatment outcomes and overall oral health [17].

In the present study, only six patients underwent DGA, with a mean interval of 11 months between SDF application and DGA referral. This finding aligns with a U.S. study suggesting that SDF may provide temporary caries control while children await DGA or become able to accept treatment under routine clinical conditions [26]. SDF may also help control disease progression and reduce pain, as suggested by a reported 80% reduction in emergency visits among children awaiting DGA following SDF application [27]. Moreover, repeated minimally invasive care may gradually improve cooperation through desensitization and behavioural adaptation, allowing future treatment without DGA [28, 29].

The experience of the paediatric dental team in managing children with limited cooperation may also have contributed to the low number of children who underwent DGA. In this study, care was provided by trained postgraduate paediatric dentistry students and specialists, with students working under specialist supervision. The observed outcomes should therefore be understood as resulting from the complete clinical pathway, including SDF, preventive care, behavioural management, improving cooperation, and the experience of the dental team, rather than SDF alone.

In Germany, nearly 80% of paediatric dentists report limited access to DGA resources [30]. Given that DGA procedures are up to eight times more expensive than preventive programmes [31], an SDF-based minimally invasive treatment pathway may represent a practical and potentially less costly management option for high-risk children. However, cost-effectiveness was not evaluated in this study and should be investigated in future research.

This study suggests that SDF may support a staged, minimally invasive treatment pathway and help avoid or postpone DGA. However, these findings should be interpreted considering certain methodological considerations. SDF enabled initial clinical caries stabilisation in many patients, providing time for their cooperation to improve and allowing simple restorative procedures to be performed later under routine clinical conditions. In our setting, the need for restorative or aesthetic treatment alone was not considered an indication for DGA. However, because some teeth subsequently received restorative treatment, the favourable outcomes should be attributed to the overall treatment pathway rather than to SDF alone. Subsequent restorative treatment could not be included analytically as a time-varying exposure. Therefore, its individual contribution to tooth-level outcomes could not be separated from that of SDF and the other components of the staged treatment pathway. The number of failure events at the tooth level was relatively small (*n* = 31) in relation to the number of covariates included in the Cox regression models, which may affect the precision of the estimated hazard ratios. The median survival time could not be estimated because fewer than half of the teeth experienced a major failure during follow-up.

In terms of study design, the study context is particularly relevant given that the only SDF-containing product currently available in Europe is not formally approved for the indication of caries arrest. As a result, retrospective analyses of routinely collected clinical data provide a pragmatic and ethically appropriate approach for evaluating the performance of SDF within the current European regulatory framework. In this setting, such real-world evidence provides valuable insights into clinical outcomes in high-risk populations where prospective controlled studies may be challenging to implement.

Overall, this study describes favourable outcomes following a staged, minimally invasive treatment pathway initiated with SDF in children with high caries risk and extensive treatment needs. A high proportion of children were managed without DGA, and more than half of the treated teeth required no subsequent restorative intervention. These findings support further investigation of SDF-based pathways as a means of bridging preventive and operative paediatric dentistry and promoting a more sustainable, lower-risk, and child-centred model of care.

## Conclusion

In this retrospective cohort, a staged, minimally invasive treatment pathway initiated with 38% SDF was associated with favourable caries-management outcomes in children with a high caries burden, most of whom were managed without DGA. Initial caries stabilisation may provide time for behavioural management, improved cooperation, and subsequent minimally invasive restorative treatment, thereby supporting the postponement or potential avoidance of DGA. However, comparative prospective studies are needed to determine the specific contribution of SDF to these outcomes

## Data Availability

Insight into the original dataset may be possible upon reasonable request addressing the corresponding author due to classified sensitive data. Prior to obtaining insight into the dataset, a security clearance approved by the Ethics Committee of Greifswald University, Germany might be required.
